# Characterization of the First Conotoxin from *Conus ateralbus*, a Vermivorous Cone Snail from the Cabo Verde Archipelago

**DOI:** 10.3390/md17080432

**Published:** 2019-07-24

**Authors:** Jorge L. B. Neves, Julita S. Imperial, David Morgenstern, Beatrix Ueberheide, Joanna Gajewiak, Agostinho Antunes, Samuel D. Robinson, Samuel Espino, Maren Watkins, Vitor Vasconcelos, Baldomero M. Olivera

**Affiliations:** 1 Department of Biology, University of Utah, 257 S 1400 E, Salt Lake City, UT 84112, USA; 2 CIIMAR/CIMAR—Interdisciplinary Centre of Marine and Environmental Research, Terminal de Cruzeiros, do Porto de Leixões, 4450-208 Porto, Portugal; 3 FECM—Faculty of Engineering and Marine Science, University of Cabo Verde, Mindelo CP 163, Cabo Verde; 4 Langone Medical Center, Department of Biochemistry and Molecular Pharmacology, New York University, New York, NY 10016, USA; 5 Department of Biology, Faculty of Sciences, University of Porto, 4169-007 Porto, Portugal

**Keywords:** conotoxin, cone snail, *Conus*, *Conus ateralbus*, Kalloconus

## Abstract

*Conus ateralbus* is a cone snail endemic to the west side of the island of Sal, in the Cabo Verde Archipelago off West Africa. We describe the isolation and characterization of the first bioactive peptide from the venom of this species. This 30AA venom peptide is named conotoxin AtVIA (δ-conotoxin-like). An excitatory activity was manifested by the peptide on a majority of mouse lumbar dorsal root ganglion neurons. An analog of AtVIA with conservative changes on three amino acid residues at the C-terminal region was synthesized and this analog produced an identical effect on the mouse neurons. AtVIA has homology with δ-conotoxins from other worm-hunters, which include conserved sequence elements that are shared with δ-conotoxins from fish-hunting *Conus*. In contrast, there is no comparable sequence similarity with δ-conotoxins from the venoms of molluscivorous *Conus* species. A rationale for the potential presence of δ-conotoxins, that are potent in vertebrate systems in two different lineages of worm-hunting cone snails, is discussed.

## 1. Introduction

The cone snails (genus *Conus*) are a biodiverse lineage of venomous predators; most species specialize in envenomating a narrow range of prey. On the basis of their primary prey, species in the genus *Conus* are divided into three broad classes, fish-hunting, snail-hunting and worm-hunting species; the great majority of *Conus* are vermivorous—worm-hunting. Pioneering studies on cone snail venoms by Endean and coworkers [1] demonstrated that the efficacy of the venom observed on particular animals could be correlated to the prey of that species. Fish-hunting cone snail venoms were highly potent on vertebrates, with worm-hunting *Conus* venoms much less so. Snail-hunting *Conus* venoms are extremely potent when tested on gastropods, but much less effective in vertebrate systems. Thus, the venom components that are highly expressed are presumably under strong selection for high potency and efficacy on molecular targets in the prey of each *Conus* species.

The initial characterization of one family of conotoxins, the δ-conotoxins, followed this general pattern. The first venom peptides of this family identified were from snail-hunting *Conus* species, such as *Conus textile* [2] or *Conus gloriamaris* [3]. Although these were highly potent when tested on molluscan systems, they were relatively inactive on vertebrates. In contrast, δ-conotoxins from fish-hunting cones, such as δ-conotoxin PVIA [4], from *Conus purpurascens*, and δ-conotoxin SVIE [5] from *Conus striatus*, were extremely potent when tested on fish or mice. 

Thus, it was a surprise to discover a δ-conotoxin from a worm-hunting species, e.g., *Conus tessulatus* and *Conus suturatus*, that was highly potent and efficacious on vertebrate systems [6,7]. Aman et al. (2015) [6] rationalized their discovery by suggesting that the ancestral worm-hunting species that gave rise to fish-hunting lineages of *Conus* had evolved a δ-conotoxin. If a K-channel blocker acting on the same circuitry were subsequently evolved, this would result in a powerful tetanic paralysis of the fish. Major lineages of fish-hunting cone snails have been shown to have both a δ-conotoxin, as well as a κ-conotoxin that blocks K channels, a combination known as the “lightning-strike cabal”. Thus, the presence of δ-conotoxins in the ancestral worm-hunting *Conus* was postulated to be critical in the shift from worm hunting to fish hunting. If this ancestor of fish-hunting lineages had indeed evolved a δ-conotoxin effective on fish, then related peptides might still be found in worm-hunting lineages descended from the same ancestral species, such as *Conus tessulatus*. 

**Figure 1:**
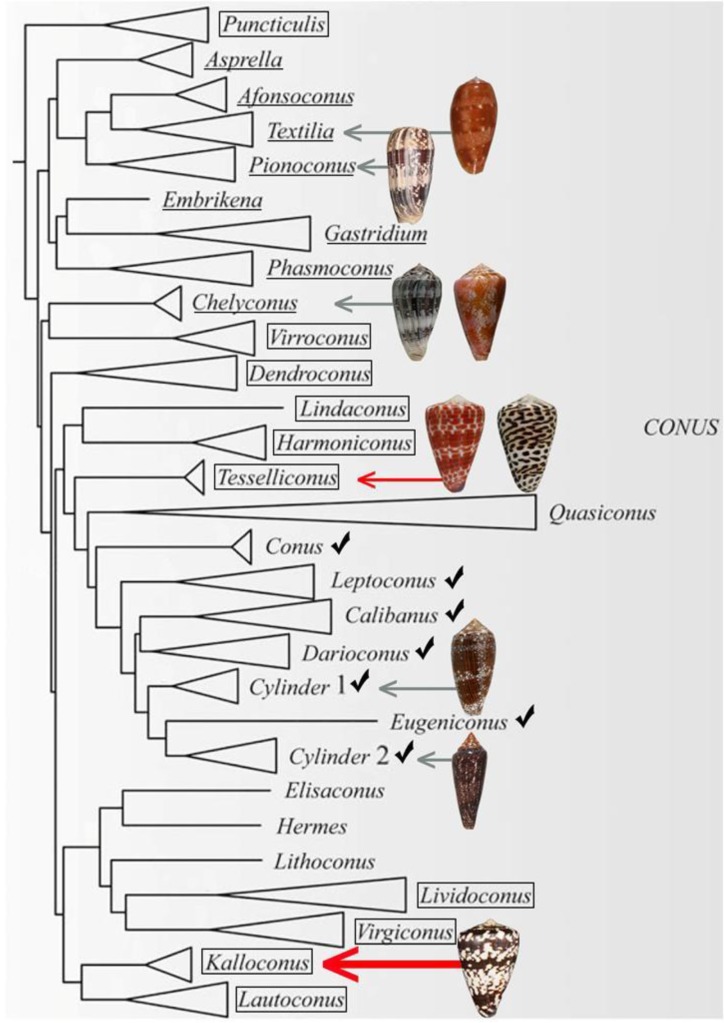
A phylogenetic tree showing the large clade of *Conus* encompassing all lineages that are fish-hunting (underlined) and snail-hunting (checked). Well-established worm-hunting lineages are boxed. δ-conotoxins have been characterized from the nine species figured, including the worm-hunting *Conus tessulatus* (red arrow). In this work, we investigated the venom of *Conus ateralbus* (shown by the thick red arrow), which is in the Kalloconus lineage. This tree was adapted with permission from Puillandre et al. (2014) [8], [*Mol. Phylogenet. Evol.*], [Elsevier Inc.], [2014]. Species discussed in this study whose shells are figured are (from top to bottom): *Conus bullatus* (Textilia); *Conus striatus* (Pionoconus); *Conus ermineus* (l) and *Conus purpurascens* (r) (Chelyconus); *Conus tessulatus* (l) and *Conus eburneus* (r) (Tesseliconus); *Conus textile* and *Conus gloriamaris* (Cylinder); *Conus ateralbus* (Kalloconus).

The present phylogenetic organization [8] of the genus *Conus* is shown in Figure 1. Fish-hunting cone snail lineages are shown underlined, and the position of *Conus tessulatus*, the worm-hunting species in the Tesseliconus lineage that yielded the δ-conotoxin described above, is shown by the red arrow. The phylogeny shown in Figure 1 is consistent with the hypothesis of Aman et al. However, there is a diversity of other worm-hunting lineages that have presumably descended from the same ancestral species, indicated by boxed lineages (mollusc-hunting lineages, marked by checks, are also predicted to have evolved from the same worm-hunting ancestor).

In this report, we demonstrate that one of the other descendant worm-hunting lineages, which is distant from Tesseliconus (the lineage that gave rise to *Conus tessulatus*), and which is presently restricted to an entirely different biogeographic range, does indeed contain a δ-conotoxin-like that is highly potent on vertebrate targets. 

**Figure 2:**
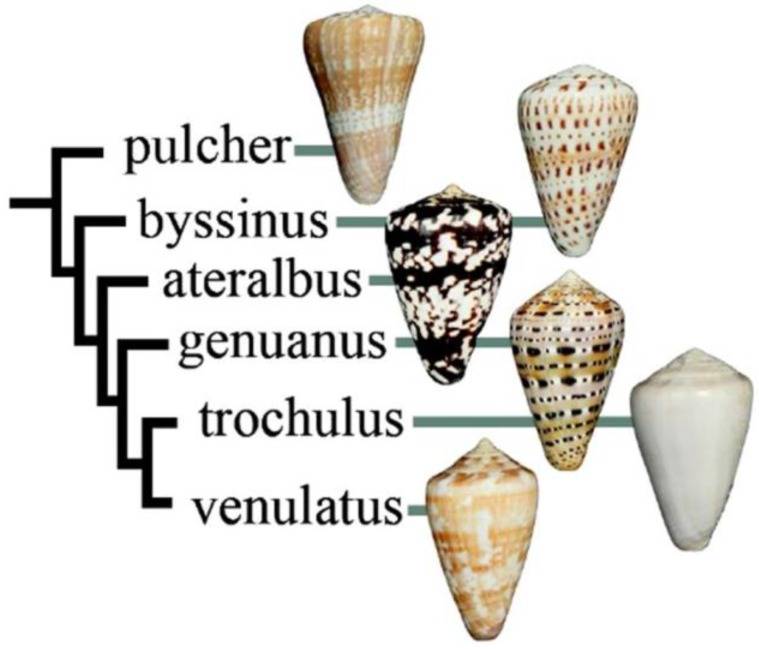
Relationship of species in Kalloconus. Most of the species are approximately the same size (ca. 40 mm), except for *Conus pulcher*, which can be very much larger (up to 230 mm). This tree was adapted with permission from Puillandre et al. (2014) [8], [*Mol. Phylogenet. Evol.*], [Elsevier Inc.], [2014].

This peptide was discovered in a lineage of West African cone snails, the subgenus Kalloconus. A phylogenetic tree showing *Conus ateralbus*, the species analyzed, and other species in the subgenus is shown in Figure 2. This is the first venom characterized from any species in the Kalloconus lineage. The subgenus Kalloconus is restricted to tropical West Africa, from the Islands of Madeira to South Angola (in contrast, Tesseliconus is only found in the Indo-Pacific). Kalloconus species comprise some of the larger *Conus* species found in the Eastern Atlantic, including *Conus pulcher*, the largest species in the entire superfamily Conoidae, growing to a length of 230 mm. Although the shells of Kalloconus species have long been used as cultural objects in northwest Africa, as well as being prized collector’s items for many centuries, little was known about their biology and some of the species in this clade have only recently been described [8]. The focus of this article is a single species of Kalloconus, *Conus ateralbus*, and a specific venom component from this species that has broader significance for toxinology. 

This is the first toxinological study on any species in the subgenus Kalloconus. We detail both the collection data and the biological observations made in the field regarding *Conus ateralbus*; some of these may apply more broadly to all Kalloconus species. In contrast to some other species in Kalloconus (e.g., *Conus pulcher* and *Conus genuanus*) that are widely distributed across the West African marine biogeographic province, *Conus ateralbus* is an endemic species in Kalloconus with the narrowest known biogeographical range. It is restricted to the west coast of the Island of Sal, in the Cabo Verde archipelago. It has been suggested that an ancestral Kalloconus from the West African coast colonized the Cabo Verde archipelago relatively recently (4.6 MYA), giving rise to at least five extant species that are endemic to Cabo Verde [9]. The specimens analyzed in this study were collected by the senior author, who also recorded field observations that provide direct evidence for the vermivory of *Conus ateralbus* (and by implication, the entire Kalloconus clade).

## 2. Results

### 2.1. Collection of Conus Ateralbus and Venom Fractionation

**Figure 3:**
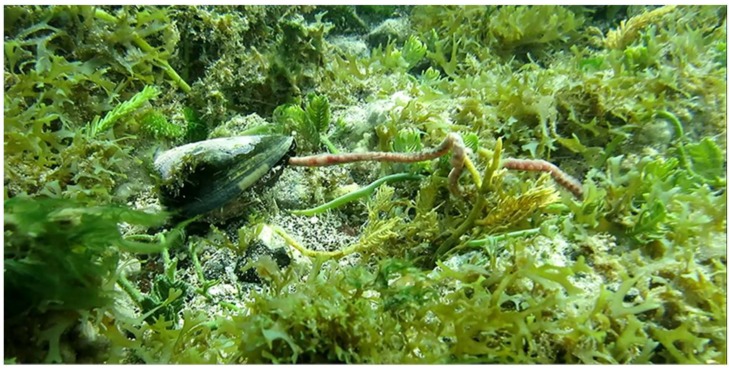
*Conus ateralbus* was observed in the field feeding on a long worm, many times the length of the predatory snail.

Specimens of *Conus ateralbus* can be reliably collected on the west coast of the Island of Sal from April to June; the field collection protocol is detailed under Methods. One specimen of *Conus ateralbus* was found consuming a polychaete worm (Figure 3). The specimen did not bury itself as it consumed the worm and it took about one hour—it was thus exposed the entire time it was feeding on its prey. As can be seen in the figure (and in the video supplied as Supplemental Material), the prey was far longer than the cone snail itself.

**Figure 4:**
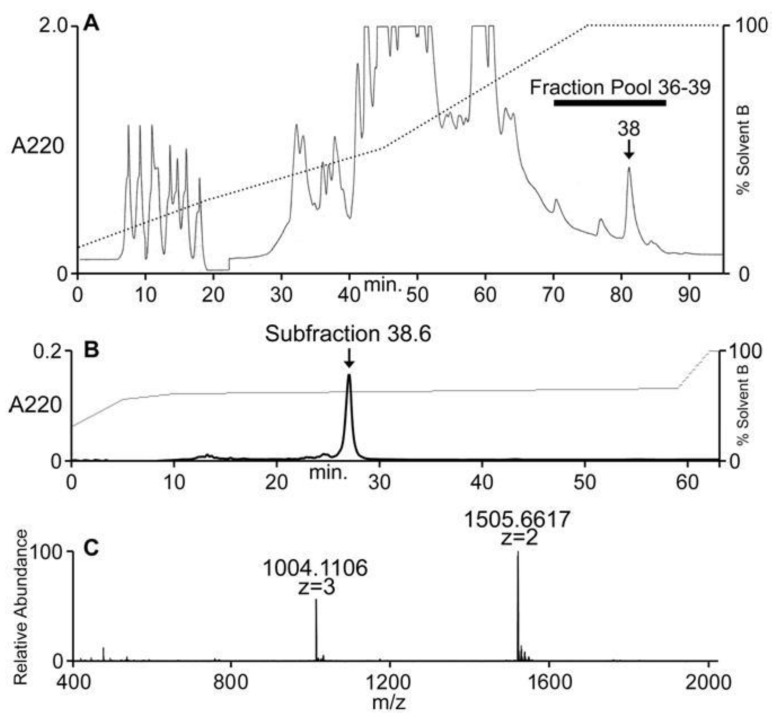
Fractionation of *Conus ateralbus* venom. The peptide characterized in this study was first detected in the pool of fractions shown, 36–39 (**A**). Further purification of the active fraction (fraction 38) yielded the chromatogram shown in (**B**). The major peak (subfraction 38.6) was both biologically active (Figure 5) and gave the results shown in (**C**) upon analysis on an Orbitrap Elite mass spectrometer at 120,000 resolution.

*Conus ateralbus* venom from many specimens was pooled and the venom extract was assayed as described under Methods. The venom extract showed activity on dorsal root ganglion (DRG) neurons and in intracranially-injected mice. The venom extract was fractionated; the HPLC chromatogram is shown in Figure 4A. Several biologically-active fractions were detected; Fraction 38, which showed activity in mice and DRG neurons, was further sub-fractionated and the biologically-active component was purified to homogeneity. The activities on DRG and mice were found in the major peak (Figure 4B); matrix-assisted laser desorption ionization mass spectrometry (MALDI-MS; linear mode) revealed this major peak to be homogeneous with a molecular mass of 3010 Da (Figure 4C). In order to determine the number of cysteine residues, the peptide was reduced with DTT and alkylated with 4-vinylpyridine; the mass increment upon pyridylethylation corresponded to the presence of six cysteine residues.

### 2.2. Conus Ateralbus Venom: Biological Activity

The HPLC fractions were assayed using calcium imaging of native DRG neurons [6,10,11] and intracranial injections on mice. The activity that caused excitatory effects on a majority of the DRG cells eluted extremely late (fraction 38), as shown in Figure 4A, suggesting the highly hydrophobic nature of the active component. Compared to control mice, the main behavioral phenotype elicited by the pool fraction, the fraction 38 and the subfraction 38.6 in mice was hypersensitivity to stimuli like touch [12].

**Figure 5:**
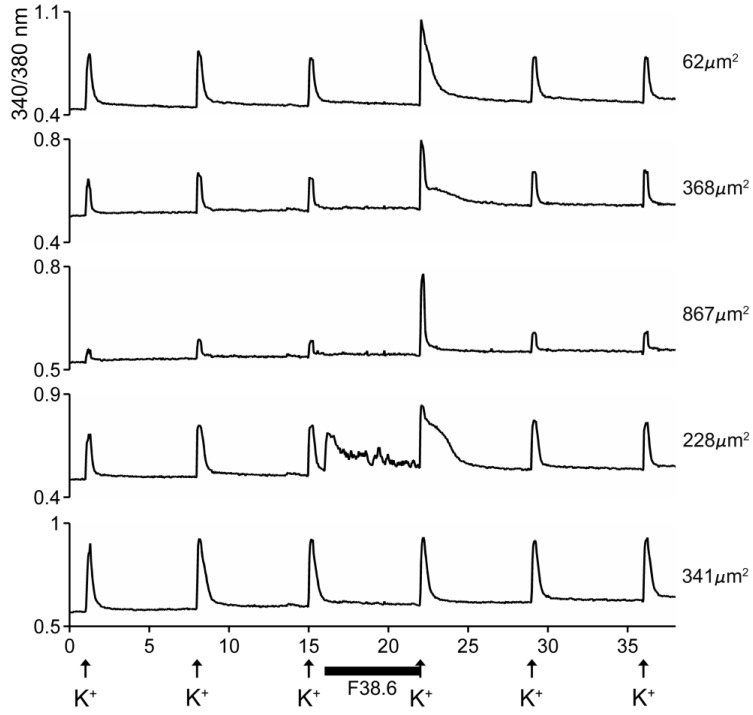
Responses of five different cells to native AtVIA (subfraction 38.6). Shown are the responses of five dorsal root ganglion (DRG) neurons (see Methods); the size of each cell is indicated. A pulse of 25 mM KCl was applied as described under Methods; the horizontal bar indicates when cells were incubated with the purified peptide. The first three cells (from top) show a change in response upon depolarization with KCl. The fourth trace shows a cell that directly increased cytosolic Ca^++^ when the peptide was added, even without KCl depolarization (approximately 26% of neurons responded in this manner) and the bottom trace shows a cell that did not respond (approximately 15% of DRG neurons were non-responsive to the peptide).

The excitatory activity that was purified and characterized affected >80% of DRG neurons. Some of the results with the purified peptide are shown in Figure 5. In the experiment shown, the cells were exposed to 25 mM KCl, which elicited an increase in cytosolic [Ca^++^]. If the cells were preincubated with the purified peptide, a large fraction of the cells responded with an increase in the Ca^++^ influx observed after the KCl pulse (first three traces in Figure 5). A small fraction of the cells responded with an increase in cytosolic [Ca^++^] even without application of KCl (4th trace, Figure 5), and a minor fraction (~15%) of the cells did not respond to preincubation with the peptide (5th trace, Figure 5). It should be noted that the first three traces show cells of different sizes, and the responses to the peptide differed in detail in these individual cells.

These results are consistent with the activity of a δ-conotoxin that inhibits the inactivation of voltage-gated Na^+^ [6].

### 2.3. Peptide Sequence Determination 

**Figure 6:**
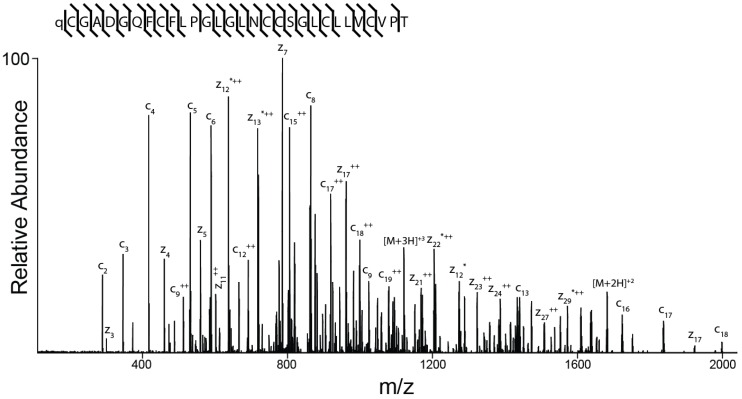
Determination of the sequence of AtVIA by tandem mass spectrometry. MS/MS-electron-transfer dissociation (ETD) spectrum of the (M + 5H) + 5 ion of qCGADGQFCF(L/I)PG(L/I)G(L/I)NCCSG(L/I)C(L/I) (L/I)VCVPT after reduction and alkylation with 2-methylaziridine acquired on the Orbitrap Elite with 15,000 resolution (@ 400 *m*/*z*). *N*-terminal fragment ions (c-type ions) are indicated by ⌉ and C-terminal fragment ions (z-type ions) are indicated by ⌊. Doubly charged ions are indicated with ++ and z ions resulting from cleavage at cysteine and loss of the cysteine side chain are indicated with *. [M + 5H]+++++•• and [M + 5H]+++++••• indicates quintuply charged precursor ions that captured 2 or 3 electrons, respectively, but have not dissociated into fragment ions. Due to space limitations, not all different charge states of already labeled peptide bond cleavages are indicated in the figure. The mass accuracy for all fragment ions is better than 15 ppm. The mass spectrometer used cannot differentiate between isoleucine or leucine and, for simplicity, leucine is used in the figure to indicate a fragment ion of mass 113.08406.

The amino acid sequence of the peptide was determined as described under Methods and is shown in Figure 6. The peptide spectrum is consistent with a 30-amino acid peptide with the following sequence: ZCGADGQFCF(L/I)PG(L/I)G(L/I)NCCSG(L/I)C(L/I)(L/I)VCVPT (where Z is pyroglutamate). It is not possible to differentiate between the isobaric amino acids isoleucine and leucine by mass spectrometry alone and this ambiguity is indicated by (L/I). 

The arrangement of the six cysteines suggests that this new peptide has an ICK structural motif and belongs to the O-superfamily, with Framework VI (C-C-CC-C-C). As shown in Table 1, the peptide shares significant sequence homology with other δ-conotoxins, e.g., δ-TsVIA, *Conus tessulatus*; δ-ErVIA, *C. eburneus*; δ-SuVIA, *C. suturatus*; δ-EVIA, *C. purpurascens*; and δ-SVIE, *C. striatus*. Together, the activity in DRG neurons and the sequence homology to δ-conotoxin shown in Table 1 are consistent with the peptide being a δ-conotoxin, i.e., inhibiting the inactivation of voltage-gated Na^+^. We, therefore, designate the peptide δ-conotoxin AtVIA.

The identity of the residues identified as (L/I) in δ-AtVIA from mass spectrometric data was resolved by screening for a genomic DNA clone encoding the peptide. The genomic DNA sequence that was obtained, which encoded AtVIA, is: catcgatcatctgtccatccatccatttcattcattcgctgccaaatggaataaatattcgcgtctctctttctgtttgtatctgacagATTGAGCAAGAAGCAGTGCGGGACTGATGGTCAGTTTTGTTTCCTACCGGGCCTTGGATTGAATTGCTGCAGTGGGCTTTGCTTAATCGTTTGCGTGCCGACATGATGTCTTCTCTTCCCCTC. Translation of the 3′ region (shown above in uppercase letters) gave: LSKKQCGTDGQFCFLPGLGLNCCSGLCLIVCVPT, and the predicted cleavage yields the peptide: QCGTDGQFCFLPGLGLNCCSGLCLIVCVPT. Q (glutamine) is a residue that is prone to spontaneously cyclize to Z (pyroglutamate) when at the *N*-terminus of a peptide [15]. It is apparent that only the (L/I) closest to the C-terminus in the sequence obtained from mass spectrometry is present as I (isoleucine), the rest of the (L/I)’s is present as L (leucine).

### 2.4. Synthesis and Folding of AtVIA[I25L;V28L;T30S] 

**Figure 7:**
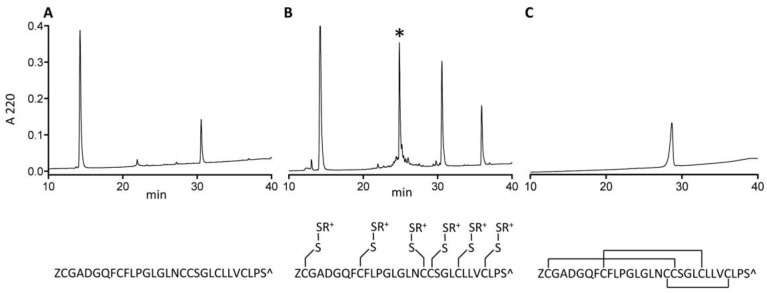
Synthesis of AtVIA[I25L;V28L;T30S]. (**A**) A crude pellet of AtVIA[I25L;V28L;T30S] was suspended in high acetonitrile content HPLC buffer and injected on C18 RP-HPLC. No peak of the desired peptide was observed. (**B**) Methane thiosulfonate bromide (MTS-ET) treatment of the crude peptide led to a S-thiocholine modified peptide (as shown below the HPLC chromatogram); the peak of the temporary S-modified peptide is indicated with an asterisk (*) on the HPLC chromatogram. (**C**) HPLC profile of the folded and purified AtVIA[I25L;V28L;T30S], with an assumed disulfide bond pattern indicated below the HPLC chromatogram.

The δ-conotoxin family is known to be difficult to synthesize and correctly fold due to their highly hydrophobic character [13,16]. While some of them are relatively easy to handle in linear form (e.g., PVIA), others are not; AtVIA[I25L;V28L;T30S], which was the peptide identified from the early sequencing results, is a good example of such behavior. When suspended in HPLC solvent with high acetonitrile content (>50%) and injected on the C18 column, no peptide peak was observed (Figure 7, Panel A). However, with methane thiosulfonate bromide (MTSET) treatment for 1 h 30 min, a major product appeared (Figure 7, Panel B). Its identity was confirmed by mass spectrometry. Methanethiosulfonate reagents (MTS-R) are known to rapidly and selectively react with cysteine residues forming mixed disulfides [17]. Thus, all 6 cysteines of AtVIA[I25L;V28L;T30S] were modified with thiocholine residues, thereby increasing overall solubility of the peptide, and making it easy to purify by reversed-phase high performance liquid chromatography (RP-HPLC). Such mixed disulfides are reversible and do not interfere with the subsequent oxidative folding reaction, which was shown before [18] for the synthesis and folding of hepcidin via S-sulfonation. Linear AtVIA[I25L;V28L;T30S] with thiocholine-modified cysteine residues folded within 4 h in a buffered solution (pH = 8.7), in the presence of 5% Tween 40 and 1:1 mixture of reduced and oxidized gluthatione with 14% yield (Figure 7, Panel C). The temporary peptide modification with MTS-R reagent can be a useful method for improving the solubility of highly hydrophobic conopeptides, in addition to already existing methods, including recently published approach utilizing an acid-cleavable solubility tag [19]. 

#### Biological Activity of AtVIA[I25L;V28L;T30S]

**Figure 8:**
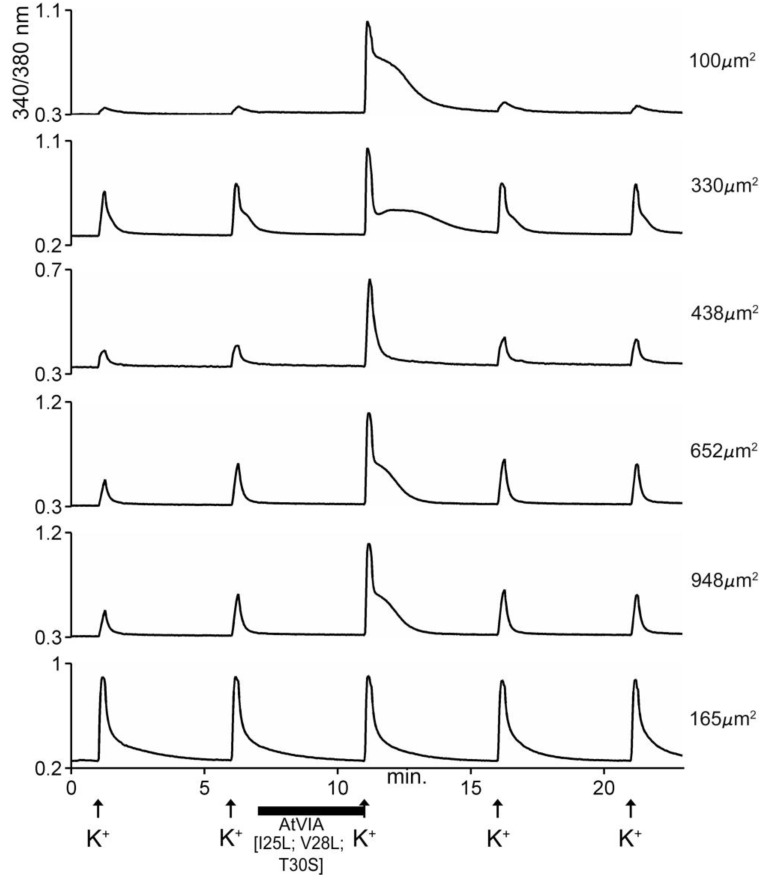
Responses of 6 different cells to AtVIA[I25L;V28L;T30S]. Shown are the responses of 6 DRG neurons, with the size of each indicated. Pulses of 25mM KCl were applied as described under Methods; the horizontal bar indicates where the cells were incubated with the peptide. The first five traces (from top) show a change in response upon depolarization with KCl (~95% of neurons responded in this manner). The bottom trace shows a cell that did not respond (~5% of DRG neurons).

The data obtained by calcium imaging in native DRG neurons in the presence of the AtVIA analog is shown in Figure 8. There is no apparent difference between the indirect effects of the native sample of AtVIA (Figure 5) and that of AtVIA[I25L;V28L;T30S]. However, the direct effects observed on the addition of the native sample of AtVIA were not observed with AtVIA[I25L;V28L;T30S]. These direct effects (Figure 5, 4th trace from the top) could be attributed to the presence of a trace of impurity in the native sample; on the other hand, the absence of these direct effects in the presence of the synthetic sample could also be attributed to any of the substitutions made on the amino acid sequence of native AtVIA.

## 3. Discussion

We report the first peptide isolated and characterized from *Conus ateralbus* venom. *Conus ateralbus* is an endemic cone snail species that belongs to the subgenus Kalloconus from the Cabo Verde Islands, an isolated archipelago in the tropical Atlantic Ocean. This species is only found on Sal Island (Calheta Funda and Mordeira Bays). The phylogenetic tree in Figure 2 shows a close relationship with two other species endemic to the Cabo Verde archipelago, *Conus trochulus* (found only in Boa Vista) and *Conus venulatus* (found on Sal, Boa Vista, Maio and Santiago) [20,21], and with *Conus genuanus* (non endemic). It was previously suggested that *Conus ateralbus* was worm hunting, based on the analysis of the radular teeth [21]; we provide direct field observations that definitively establish *Conus ateralbus* as a worm hunter (Figure 3). Thus, it is likely that all Kalloconus are worm hunting, since specific clades in the genus *Conus* generally sort out on the basis of their primary prey. It is notable that in the phylogenetic tree in Figure 1, there are clusters of worm-hunting lineages that are well separated from each other. *Conus tessulatus*, in the subgenus Tesseliconus clusters with Harmoniconus and Lindaconus. In contrast, Kalloconus clusters with Lautoconus (also West African) and less closely with Virgiconus and Lividoconus (both Indo-Pacific). In the present work, we establish the presence of δ-conotoxins that act on vertebrate Na channels in the two divergent clusters of worm-hunting clades, Kalloconus and Tesseliconus. These δ-conotoxins vary greatly with respect to their sequence similarities (see Table 1A), in a manner concordant with the phylogenetic tree shown in Figure 1. 

The *Conus ateralbus* venom peptide, δ-conotoxin-like AtVIA (δ-AtVIA; GenBank accession number is MH025915), was purified to homogeneity and biochemically characterized. A clone was identified in order to sort out the I/L uncertainty that is inherent in sequence data obtained by mass spectrometry. Despite the conservative amino acid substitutions on the C-terminal region based on the early mass spectrometric sequencing data, exhibited indirect effects on mouse DRG responses to depolarization with KCl, which were identical to those of the native δ-AtVIA and δ-TsVIA [6].

Table 1 shows δ-AtVIA and other δ-conotoxin sequences from various subgenera of *Conus*, including fish-hunting, snail-hunting and worm-hunting lineages. As shown in Table 1A, there are conserved sequence features in venom peptides (highlighted in yellow; 52–59% of conserve sequence between δ-AtVIA petide, δ-TsVIA, δ-ErVIA and δ-SuVIA) from the two worm-hunting and three fish-hunting subgenera identified that are not shared by the peptides from snail-hunting species. The conserved sequence features in the fish-hunting and worm-hunting cone snail δ-conotoxins shown in Table 1 are presumably important for targeting these peptides to vertebrate Na channels. In contrast, the sequences of δ-conotoxins from snail-hunting species do not share these consensus sequence features and are not broadly effective on vertebrate Na channels.

It should be noted that although there are conserved sequence features between δ-conotoxins from worm-hunting and fish-hunting species, there are also systematic subgeneric differences between groups of peptides. Thus, the peptide from *Conus ateralbus* is much more divergent (14AA differences) from the three sequences from the three Tesseliconus species (*Conus tessulatus*, *Conus eburneus*, and *Conus suturatus* that differ by 1-4AA from each other). However, δ-conotoxin structures provide amino acids critical for the activity of conotoxins, and it was demonstrated that homologous sequences with large hydrophobic amino acids of δ-conotoxin are very relevant for activity on vertebrate Na channels [13]. Thus, three large hydrophobic amino acids (F, L and I) are conserved in all sequence of the peptides AtVIA, TsVIA, SuVIA and ErVIA, on the first, second and fourth intercysteine loops [6,7]. δ-conotoxin has some other characteristic proprieties, as the HPLC similar profile of the longest retention time and a well-shaped peak in reverse-phase column. In addition, the worm-hunting peptides can be separated from those from fish-hunting cone snail venoms by examining the loop between the second and third Cys residues in these peptides. In all the δ-conotoxins from fish-hunting cones, the third amino acid in this loop is always a positively charged residue (i.e., K in the Chelyconus sequences, H in the Pionoconus peptide and R in the Textilia peptide); this positively charged residue is missing from the peptides from worm-hunting *Conus*. Furthermore, although the proline residue is conserved in this loop, it is post-translationally modified to hydroxyproline in all of the sequences from fish-hunting species but is unmodified in the sequences from worm-hunting species. The differences between the peptides derived from fish-hunting versus worm-hunting *Conus* suggest that the spectrum of voltage gated sodium channels might systematically differ between the two classes of peptides. If this were the case, then δ-conotoxins, such as the peptide we have characterized here from *Conus ateralbus*, may prove to be useful pharmacological reagents for differentiating between the various molecular subtypes of voltage gated sodium channels. 

The sequence conservation that is highlighted in Table 1 is consistent with the hypothesis that the last common ancestor of the four fish-hunting species, whose shells are shown in the figure (i.e., *Conus purpurascens*, *Conus ermineus*, *Conus striatus*, and *Conus bullatus*) and the four worm-hunting species (i.e., *Conus eburneus*, *Conus tessulatus*, *Conus suturatus* and *Conus ateralbus*) had already evolved a δ-conotoxin with these consensus sequence features as predicted by the hypothesis detailed by Aman et al., (2015) [6] for the molecular events that accompanied the prey shift from worm hunting to fish hunting. Recently, it was demonstrated that Cone snails specialized in two different evoked venom, the predation region and defense region on the venom gland [22]. Worm-hunting cone snails, as *Conus suturatus*, specialized defensive envenomation strategy in the proximal regions of the venom duct. δ-conotoxins in the defense-evoked of the *Conus* venom gland contribute to the understanding of the evolution from worm-hunting to fish-hunting cone snails [7,22].

As shown in Table 1, two snail-hunting δ-conotoxins from the subgenus Cylinder, δ-TxVIA and δ-GmVIA, do not share these consensus sequence features. Since these snail-hunting species are also descended from the last common ancestor referred to above—this would, therefore, appear to be inconsistent with the hypothesis of Imperial et al., (2007) [23] and Aman et al., (2015) [6].

There are two possible explanations for the observed lack of conservation in δ-conotoxins from the snail-hunting *Conus* species. First, δ-conotoxins play a major role in prey capture; a snail hunter has the biological problem of keeping the envenomated snail outside its shell. A predator striking a snail would elicit the automatic response of the prey withdrawing deeply into the shell; snail-hunting cone snails have no way of breaking the shell of their potential prey. The δ-conotoxins are key to activating motor circuitry so that immediately after envenomation, the body of the prey is extended outside the shell accompanied by spastic, uncoordinated movements, and is unable to withdraw into its shell [24]. This then allows further injection of venom, providing continuous access to the soft parts of the prey without having to break the shell. Thus, the δ-conotoxins from molluscivorous *Conus* that have been purified and characterized seem likely to play a role in prey capture, so it would be expected that these are targeted to molluscan sodium channels and are divergent in sequence from the ancestral δ-conotoxin. Additionally, because the prey of snail-hunting cones has a hard shell, retention of a peptide that deters fish competitors may no longer be required. Thus, these explanations rationalize why δ-conotoxin sequences from *Conus* textile and *Conus* gloriamaris do not have the consensus features of the other δ-conotoxins in Table 1, and why these are not active on vertebrate voltage-gated sodium channels. 

## 4. Materials and Methods 

### 4.1. Field Collection and Venom Extraction 

*Conus ateralbus* specimens were collected in the Calheta Funda Bay, Sal Island, in shallow water (around 2 m deep) in 2013. The specimens were collected in one day, kept alive in seawater and preserved at −20 °C at the end of the day. The venom duct was dissected from each frozen specimen. Venom was obtained from ducts immediately after dissection by placing each duct on an ice-cold metal spatula; the venom was squeezed out using an Eppendorf pipette tip and was lyophilized and stored at −80 °C. Crude venom extracts were prepared using 40% (*v*/*v*) CH3CN/water acidified with 0.1% (*v*/*v*) trifluoroacetic acid (TFA). A 36.5 mg portion was resuspended in 15 mL of 40% acetonitrile and 0.1% trifluoroacetic acid (TFA) using a vortex mixer for 2 × 1 min with an interval of 3 min, homogenized in a Wheaton homogenizer and centrifuged in a Beckman Avanti centrifuge (F650 rotor) for 15 min at 13.650 rpm, at 4 °C. The supernatant was centrifuged again to remove all residual particles.

### 4.2. Venom Fractionation 

Crude extract from 36.5 mg of venom was fractionated by reversed-phase high performance liquid chromatography (RP-HPLC) using a C18 Vydac 218TP101522 preparative column. Elution was done at a flow rate of 7 mL/min and a gradient ranging from 10% to 30% of solvent B in 20 min, 30% to 50% in 25 min, 50% to 100% in 30 min, and 100% for 15 min. Solvent B was 90% (*v*/*v*) CH3CN in 0.1% (*v*/*v*) aqueous TFA, and solvent A was 0.1% (*v*/*v*) TFA in water. The subfractionation of the active fraction 38 was done by RP-HPLC using a C18 Vydac monomeric 238EV54 column. The absorbance was monitored at 220 and 280 nm.

### 4.3. Mass Spectrometry and Sequence Determination

The crude HPLC fractions were analyzed using matrix-assisted laser desorption ionization (MALDI) mass spectrometry. The AtVIA sample (subfraction 38.6, Figure 4B) was dissolved in 100 µL of 0.5% acetic acid; 10 µL of the solution were desalted using POROS R2 beads [25]. An aliquot of the unreduced sample was loaded onto a 0.2 × 25 cm Pepswift EasySpray column. The sample was eluted at a flow rate of 1 µL/min with a gradient of 0–100% of Solvent C (90% (*v*/*v*) CH3CN in 0.5% acetic acid) in 20 min, a spray voltage of 2.5 kV on an Easy nLC-1000 nanoUHPLC coupled to an Orbitrap Elite mass spectrometer. MS1 scans were acquired at 120,000 resolution (@ 400 *m*/*z*). For MS2, the most abundant precursor was isolated and fragmented using ETD at 15,000 resolution (@ 400 *m*/*z*) and 60 msec ion reaction time. 

The fraction (subfraction 38.6, Figure 4B) was reconstituted in 100 μL of 0.5% acetic acid. For determination of the accurate mass of the peptide (better than 10 ppm) an aliquot of this fraction (1%) was loaded onto a 200 µm × 25 cm Pepswift EasySpray column using the autosampler of an Easy nLC-1000 nano-HPLC coupled to an Orbitrap Elite mass spectrometer. The sample was eluted using a flow rate of 1ul/min with a gradient of 0–100%B (solvent A = 0.5% acetic acid, solvent B = 90% acetonitrile in 0.5% acetic acid (*v*/*v*)) in 20 min and a spray voltage of 2.5 kV. MS1 scans were acquired at 120,000 resolution (@ 400 *m*/*z*). For de novo sequence determination another aliquot of the sample was dried in the speedvac and subsequently reduced and alkylated in vapor using 1% 2-methylaziridine and 2% trimethylphosphine in 50% acetonitrile and 100 mM ammonium bicarbonate (pH 8.4) for 90 min at RT. Alkylation vapor was removed and the sample was reconstituted in 0.5% acetic acid. Aliquots of the now reduced and alkylated samples was loaded onto a 200 m × 25 cm Pepswift EasySpray column using the autosampler of an Easy nLC-1000 nano-HPLC coupled to an Orbitrap Elite mass spectrometer as described above. The gradient was 0–50%B in 50 min. MS1 scans were acquired at 120,000 resolution (@ 400 *m*/*z*). MS2 was acquired on the top 5 precursors that carry at least 4 charges using the following settings: 4 microscans, 3 *m*/*z* isolation window, target value of 1e4 ions. Each precursor was subjected to ETD and HCD fragmentation using the following conditions: 15,000 resolution (@ 400 *m*/*z*), 30 s dynamic exclusion, ETD using 60 ms ion reaction time with supplemental activation, HCD using 27% normalized collision energy. The sequence was obtained by manual de novo sequencing. The measured mass deviates from the theoretical mass by 5.5ppm and is within the mass error of the instrument. 

#### Preparation of Genomic DNA and Characterization of Clones Encoding AtVIA 

Genomic DNA was prepared from 20 mg of *Conus ateralbus* venom duct using the Gentra PUREGENE DNA Isolation Kit (Gentra Systems, Minneapolis, MN, USA) according to the manufacturer’s standard protocol. *Conus ateralbus* genomic DNA (10 ng) was used as a template for polymerase chain reaction (PCR) with oligonucleotides corresponding to the conserved intron and 3′ UTR sequences of previously isolated δ-conotoxin prepropeptides [26]. The resulting PCR product was purified using the PureLink PCR Purification Kit (Life Technologies, Carlsbad, CA, USA) following the manufacturer’s suggested protocol. The eluted DNA fragment was annealed to pNEB206A vector and the products were transformed into competent Esherichia coli DH5α cells, using the USER^®^ Friendly Cloning Kit (New England Biolabs, Inc., Ipswich, MD, USA) following manufacturer’s suggested protocols. The nucleic acid sequence of this δ-conotoxin-encoding clone was determined at the Core Sequencing Facility, University of Utah, USA, following the ABI automated sequencing protocol initiated by the M13 universal reverse primer. 

### 4.4. In Vivo Assay and Calcium Imaging Assay on DRG 

Each dried aliquot of HPLC fraction pools or individual fractions was resuspended in 12 µL of normal saline solution (NSS, 0.9% NaCl). Mice (male and female, were 15 days old, 6–8 g of body weight) were intracranially injected using a 0.3 mL insulin syringe (equivalent of 4 μg/μL); the same volume of NSS alone was injected in control mice. After the injection of each sample, the peptide-injected mice were observed side by side with NSS-injected controls, all mice were placed in separate cages for at least 1 h [27]. 

Lumbar dorsal root ganglia (DRG) were dissected from wild type C57/BL6 mice, dissociated, pooled and cultured overnight for calcium imaging experiments, following previously described protocols [10]. Cells were loaded with Fura-2-AM dye one hour before the experiment. During the experiment, the dye inside the cells was excited alternately with 340 nm and 380 nm light and the ratio of the emissions at 510 nm from both excitations was measured. The ratio of the fluorescence intensity was considered indicative of intracellular calcium concentration. A solution of 25 mM KCl was applied for 15 s every seven minutes to induce neuronal depolarization. After the third KCl pulse, venom extract or HPLC fractions were applied and the effects on intracellular calcium levels, before, during and after depolarization, were monitored. Experimental protocols involving live animals were approved by the Institutional Animal Care and Use Committee of the University of Utah.

### 4.5. Peptide Synthesis 

Based on the early sequencing results, AtVIA[I25L;V28L;T30S], a peptide with the following sequence: ZCGADGQFCFLPGLGLNCCSGLCLLVCLPS-OH (Z = pyroglutamate) was synthesized at 50-µmol scale using an AAPPTec Apex 396 synthesizer (AAPPTec, LLC, Louisville, KY, USA) using standard solid-phase Fmoc (9-fluorenylmethyloxycarbonyl) protocols. Fmoc-protected amino acids were purchased from AAPPTec. The peptide was assembled on pre-loaded Fmoc-L-Ser(tBu)-Wang resin (substitution, 0.53 mmol/g; Peptides International Inc., Louisville, KY, USA). Side-chain protection for each corresponding amino acid was as follows: Asp, O-tert-butyl (OtBu); Ser, tert-butyl (tBu); and Asn, Gln and Cys, trytl (Trt). Coupling of each amino acid was achieved using 1 equivalent of 0.4 M benzotriazol-1-yl-oxytripyrrolidinophosphonium hexafluorophosphate (PyBOP) and 2 equivalents of 2 M *N*,*N*-diisopropylethyl amine (DIPEA) in *N*-methyl-2-pyrrolidone (NMP). The amino acid amounts used were at ten-fold excess (60 min coupling). Fmoc-protecting groups were removed by a 20-min treatment with 20% (*v*/*v*) piperidine in dimethylformamide (DMF).

#### 4.5.1. AtVIA[I25L;V28L; T30S] Cleavage, Derivatization and Purification 

The peptide was cleaved from 100 mg of resin by treatment with Reagent K (TFA/H_2_O/phenol/thioanisole/1,2-ethanedithiol; 82.5/5/5/5/2.5 by volume). After 2.5 h, the crude peptide was separated from the resin by vacuum-filtration. The cleavage product was precipitated in cold methyl-tert-butyl ether (MTBE) and subsequently washed one more time with MTBE. The crude peptide was suspended in 50% (*v*/*v*) CH3CN in 0.01% aqueous TFA and treated with 55 mg of [2-(Trimethylammonium)ethyl] methane thiosulfonate bromide (MTSET). The pellet was still present in the solution after 30 min, so another portion of MTSET was added (~40 mg) and it was allowed to react for an additional 1h. The modified peptide was then purified by reversed-phase (RP) HPLC in a Vydac C18 semi-preparative column (218TP510, 250 mm × 10 mm, 5 µm particle size) over a linear gradient ranging from 20% to 50% of solvent B in 30 min with a flow rate 4 mL/min. The peptide was quantified by comparing the peak area obtained by analytical RP-HPLC to that of a known amount of a reference peptide, At6A[F8Y;V28L;T30S]. Out of 100 mg cleaved resin, ~500 nmol of thiocholine-modified linear peptide was obtained. The identity of the linear peptide was confirmed using ESI MS: calculated: [M+] = 3717.75, obtained: [M+] = 3717.67.

#### 4.5.2. Oxidative Folding of At6A[I25L;V28L;T30S] 

The linear, thiocholine-modified peptide (100 nmol) was re-suspended in 50% (*v*/*v*) CH3CN in 0.01% aqueous TFA and added to a solution made up of 4 mL of 0.2 M Tris-HCl—2 mM EDTA pH 8.7, 0.4 mL of (5%) Tween 40, 1.6 mL of a 1:1 mixture of 10 mM GSSG and 10 mM GSH and 1.5 mL of water. Folding reaction was conducted for 4 h at room temperature and quenched by acidification using 8% (*v*/*v*) formic acid. Peptide was purified by RP-HPLC on the C18 semipreparative column using two different gradients: 35% to 95% change of solvent B in 15 min (4%/min of gradient change, 4 mL/min of elution rate) and 35% to 95% change of solvent B in 30 min (2%/min; 4 mL/min). The identity of the peptide was confirmed by MALDI-TOF mass analysis; calculated [M + H]^+^: 3008.29 Da, observed: [M + H]^+^: 3008.33 Da, but the desired mass was represented by a minor peak. The major peaks observed were: 3030.33 Da and 3046.30 Da which correspond to: [M + Na]^+^ (calculated: 3030.27 Da) and [M + K]^+^ (calculated: 3046.25 Da), respectively. There were also masses ranging from 1300 to 1500 Da, indicating traces of Tween (used in the folding reaction) in the sample. The peptide was quantified using amino acid analysis. Out of 1200 nmols of the linear peptide, 174 nmols of the desired folded peptide was obtained.

## 5. Conclusions

In this work, we report the first venom peptide characterized from any species in the subgenus Kalloconus, δ-conotoxin-like AtVIA from *Conus ateralbus*, an endemic cone snail species from the Cabo Verde Islands. δ-like AtVIA has homology with δ-conotoxins from worm-hunting species in the subgenus Tesseliconus, including conserved sequence elements shared with δ-conotoxins from fish-hunting *Conus*. We provide direct field observations (*Conus ateralbus* filmed eating a worm) that definitely establish *Conus ateralbus* as a vermivorous *Conus* species. The presence of δ-conotoxins that act on vertebrate Na+ channels has thus been established in two divergent worm-hunting clades. The results are consistent with the hypothesis that certain worm-hunting *Conus* evolved δ-conotoxins that act to probably deter competitors in a defensive envenomation strategy, and that this may have been an intermediate evolutionary step in the shift to fish prey within the genus *Conus*.

## Figures and Tables

**Table 1:** Comparison of δ-AtVIA with δ-conotoxins from various *Conus* species.

Subgenus (Prey)	-Conotoxin	*Conus* Species	Sequence	Ref.
A.				
*Tesseliconus* (worm)	-TsVIA	*C. tessulatus*	**C**AAFGSF**C**GLPGLVD--**CC**SGR**C**FIV**C**LL	[6]
*Tesseliconus* (worm)	-ErVIA	*C. eburneus*	**C**AGIGSF**C**GLPGLVD--**CC**SGR**C**FIV**C**LP	[6]
*Tesseliconus* (worm)	SuVIA	*C. suturatus*	**C**AGIGSF**C**GLPGLVD--**CC**SDR**C**FIV**C**LP	[7]
*Kalloconus* (worm)	-AtVIA	*C. ateralbus*	Z**C**GADGQF**C**FL-PGLGLN**CC**SGL**C**LIV**C**VPT	**This work**
B.				
*Chelyconus* (fish)	PVIA	*C. purpurascens*	EA**C**YAOGTF**C**GIKOGL---**CC**SEF**C**LPGV**C**FG	[4]
*Pionoconus* (fish)	SVIE	*C. striatus*	DG**C**SSGGTF**C**GIHOGL---**CC**SEF**C**F-LW**C**ITFID	[5]
*Textilia* (fish)	-BuVIA	*C. bullatus*	DE**C**SAOGAF**C**LIROGL---**CC**SEF**C**F-FA**C**F	[13]
*Kalloconus* (worm)	AtVIA	*C. ateralbus*	Z**C**GADGQF**C**FL-PGLGLN**CC**SGL**C**L-IV**C**VPT	**This work**
*Tesseliconus* (worm)	TsVIA	*C. tessulatus*	**C**AAFGSF**C**GL-PGLVD-**CC**SGR**C**F-IV**C**LL	[6]
*Tesseliconus* (worm)	ErVIA	*C. eburneus*	**C**AGIGSF**C**GL-PGLVD-**CC**SGR**C**F-IV**C**LP	[6]
*Tesseliconus* (worm)	SuVIA	*C. suturatus*	**C**AGIGSF**C**GL-PGLVD-**CC**SDR**C**F-IV**C**LP	[7]
*Cylinder* (snail)	TxVIA	*C. textile*	W**C**KQSGEM**C**NLLDQN---**CC**DGY**C**IVLV-**C**T	[14]
*Cylinder* (snail)	GmVIA	*C. gloriamaris*	VKP**C**RKEGQL**C**DPIFQN---**CC**RGWN**C**VLF-**C**V	[3]

## Supplementary Materials

The following are available online at http://doi.org/10.5281/zenodo.3233739, Video S1: Conus ateralbus eating polychaete worm.
